# Stars and steam engines: To what extent do thermodynamics and statistical mechanics apply to self-gravitating systems? 

**DOI:** 10.1007/s11229-018-02032-5

**Published:** 2018-11-30

**Authors:** Katie Robertson

**Affiliations:** Department of Philosophy, University of Birmingham, Birmingham, B15 2TT UK

**Keywords:** Statistical mechanics, Thermodynamics, The thermodynamic limit, Self-gravitating systems, Reduction

## Abstract

Foundational puzzles surround (Newtonian) gravitational thermal physics—a realm in which stars are treated as akin to molecules in a gas. Whether such an enterprise is successful and the domain of thermal physics extends beyond our terrestrial sphere is disputed. There are successes (such as the collisionless Boltzmann equation) and paradoxical features (such as the ‘gravothermal catastrophe’). Callender (Found Phys 41(6):960–981, 2011) advocates reconciling the two sides of the dispute by taking a broader view of thermodynamics. Here I argue for an alternative position: if we are careful in distinguishing statistical mechanics and thermodynamics, then no reconciliation is required. Both sides can live in harmony because whilst statistical mechanics applies, thermodynamics does not. This state of affairs—the applicability of statistical mechanics without the emergence of thermodynamic behaviour—can be explained in terms of an infamous infinite idealisation: the thermodynamic limit.

## Introduction

The foundations of thermal physics are riddled with controversy. The keystone of the philosophical debate is the old issue of whether thermodynamics (TD) reduces in some appropriate way to statistical mechanics (SM). This issue involves analysing the thermodynamic limit (i.e. roughly the limit of an infinite number of microconstituents); and so leads immediately to the topic of infinite idealisations. Namely: how should we understand this limit given that any physical system to which we successfully apply thermodynamics and/or statistical mechanics contains in fact a finite number of atoms (or other microconstituents)? The debate in thermal physics has centred around phase transitions. The SM description of phase transitions requires the thermodynamic limit, unlike the TD description, which seemingly makes the TD description superior and so—some argue—non-reducible to SM. In this paper, I consider a different case in thermal physics where, I will argue, statistical mechanics has the upper hand. This case further differs from the usual phase transitions case: I will argue that the philosophical interest of this field of physics, and the light it sheds on the thermodynamics/statistical mechanics relation, turns on the fact that here, the thermodynamic limit does *not* exist.

This field is often called ‘gravitational thermal physics’—and so the tangles of thermal physics reach beyond our terrestrial sphere. But even if we set aside black holes, the claim that thermal physics successfully applies to *Newtonian* astrophysical contexts has been disputed. Such an enterprise involves applying the ideas of thermal physics to vast collections of stars: both globular clusters with ca. $10^5$ stars and galaxies with ca. $10^{11}$ stars. The key idea is to think of such a collection as like a gas: just as the molecules in a gas are its microconstituents, the stars in such a collection are *its* “microconstituents”. This is obviously a very striking, indeed bold, idea: both physically and philosophically. Physically, because we expect disanalogies between the idealisations made for a collection of molecules and those made for a collection of stars. In particular, stars interact by gravity, which is systematically set aside in terrestrial applications of thermal physics. Philosophically, because our epistemic access to (our warrant for believing in) molecules and stars are so very different. Stars are epitomes of the observable; since the ancients turned their eyes heavenwards, we have believed in them—though of course what we have believed *about* them has altered immensely since ancient times, especially since 1850 with the application of spectroscopy to starlight through to today’s stunning observational knowledge of stars’ lifecycles. This philosophical disanalogy between molecules and stars will play out in what follows, especially in connection with (1) the relationship of thermodynamics to statistical mechanics and (2) Einstein’s distinction between constitutive and principle theories. And as we will see, the question of the existence and the nature of the thermodynamic limit—the infinite idealisation of infinitely many stars—will be central.

Whilst such philosophical and physical disanalogies abound, ultimately the question is whether (Newtonian) gravitational thermal physics is a successful enterprise. Thus Callender asks whether “the stars in such systems or even the galaxies themselves, when idealised as point particles, admit a thermodynamic description” (Callender 2010, p. 44). Does thermal physics apply to these Newtonian self-gravitating systems? Is this an extension of the domain of applicability? Indeed, does this case give further weight to the idea that thermodynamics is universal?

On the one hand, it seems that thermal physics applies to self-gravitating systems (SGS). For instance, in certain circumstances the evolution of the distribution of stars in a galaxy can be modeled using the collisionless Boltzmann equation or the Fokker-Planck equation (see e.g. Binney and Tremaine 1987). On the other hand, self-gravitating systems exhibit many unusual features, sometimes called the ‘gravitational paradoxes’, as discussed in Sect. 2.

There is a prima facie dispute in the scientific community. Some express Optimism over the applicability of thermal physics: “Statistical mechanics of gravitating systems is a controversial subject. However, our modern understanding of statistical mechanics and thermodynamics does handle gravitational interactions rigorously with complete satisfaction” (Kiessling 1999, p. 545). Other express Pessimism: “[Thermodynamics] is essentially a human science; it started with steam engines and went on to describe many physical and chemical systems whose size is of the order of a metre. They clearly are inapplicable to the solar system or to galaxies. Clearly classical thermodynamics is not a useful branch of science in cosmology; we have extrapolated too far from its human-sized origins” (Rowlinson 1993, p. 873).

Of course, one might be tempted to ‘hedge your bets’ and claim that whilst gravitational thermal physics has some successes, this success is qualified by the paradoxes. That is, one might claim, as is often the case with optimism and pessimism, that there are shades of grey: the truth lies in between.

But I think we can do better than merely hedging our bets in this dispute. My goal in this paper is to make peace between the Optimists and the Pessimists, by deflating the debate between them. I argue that: if we are careful in distinguishing statistical mechanics and thermodynamics, then no reconciliation is required. Both sides can live in harmony because whilst statistical mechanics applies, thermodynamics does not.

This position differs from Callender (2011), who brought this dispute to the attention of the philosophy of physics community (Callender 2010). He notes the successful features emphasised by the Optimists, whilst not minimising the difficult features that a Pessimist might stress. But—motivated by his broader position in the foundations of thermal physics, namely: we should not take thermodynamics too seriously and so advocates a more flexible, and so more liberal, view of thermodynamics—Callender subscribes to a (cautious) Optimism. That is, he holds that the problems facing SGS do not “spell the end for gravitational equilibrium thermodynamics” (Callender 2011, p. 962).

A disclaimer at the outset: my reply to Callender will not hinge on bringing new physics to bear on the dispute, but rather a different perspective on the foundations of thermal physics. Thus the main message will be: the example of self-gravitating systems need not necessitate having a broader or more flexible view of thermodynamics.

In Sect. 2, I recapitulate Callender’s discussion of the thermal physics of self-gravitating systems: the difference from ordinary systems, the successes and the unusual ‘gravitational paradoxes’. Section 3 outlines my strategy of delineating SM and TD, and connects such a strategy to the reduction debate, and to Callender’s position. In Sect. 4, I argue that thermodynamics does not apply to self-gravitating systems. Section 5 outlines the extent to which statistical mechanics applies. Thus, my verdict on the dispute is that there is (to an extent) a statistical mechanical description of SGS, even though no thermodynamic behaviour emerges. Section 6 sketches an explanation of why thermodynamics and statistical mechanics come apart in this case. This explanation will hinge on the thermodynamic limit, and so I also outline the connections to the wider debate about the role of the thermodynamic limit in the relationship between SM and TD. Section 7 concludes.

## Newtonian gravity weighs in

In this section, I first review how incorporating gravity changes the physics of thermal systems and discuss the type of systems well-approximated by this treatment. I then outline two examples of successful evolution equations. Finally, I discuss some of the ‘paradoxical’ features.

### How the situation changes with gravity

**Fig. 1:**
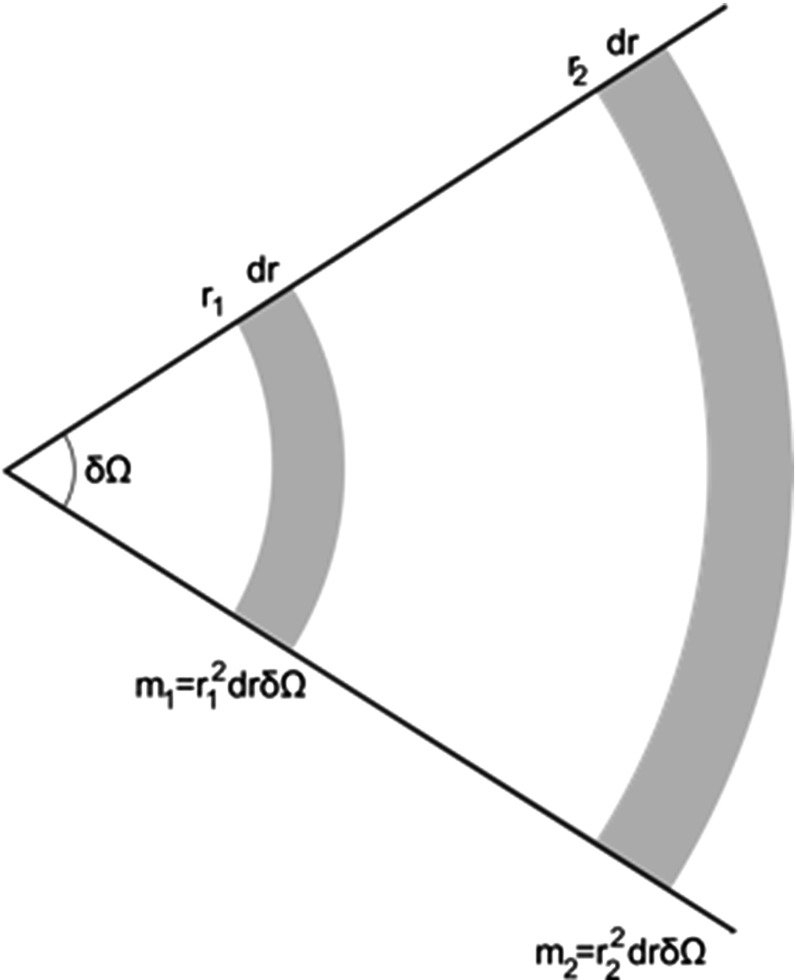
Diagram from Callender (2010) (adapted from Binney and Tremaine). In a collisionless system with constant density of stars, the force exerted on a star at the apex is the same from the band of stars $r_1$ as from the band of stars at $r_2$

Gravitational forces are negligible in the terrestrial thermal systems with which we are familiar. But in extraterrestrial systems such as galaxies, this assumption is of course no longer justified. Unlike the local collisions and forces in an ideal gas, the gravitational force is *long-range*; the range of the dominant interaction is large relative to the spatial size of the system. Consequently, the forces on a given star are not only due to its nearest neighbours, but include a contribution from the large scale structure of the stellar system. Indeed, if the density of stars is spatially constant (cf. Fig. 1), the gravitational force exerted on a given star (by the rest of the stellar system) at the apex is the same from the patch of stars of solid angle $d\Omega$ surrounding it at distance $r_1$, as from the patch at distance $r_2$. Clearly if the distribution of stars were exactly spherical, there would be no net force on this star. However, if the density of stars falls off more slowly in one direction, then only this very global feature of the entire stellar system will be responsible for the force on our star. This contrasts sharply with the forces experienced by a molecule in gas, which come only from its nearest neighbours and thus is a much more local feature.

**Fig. 2:**
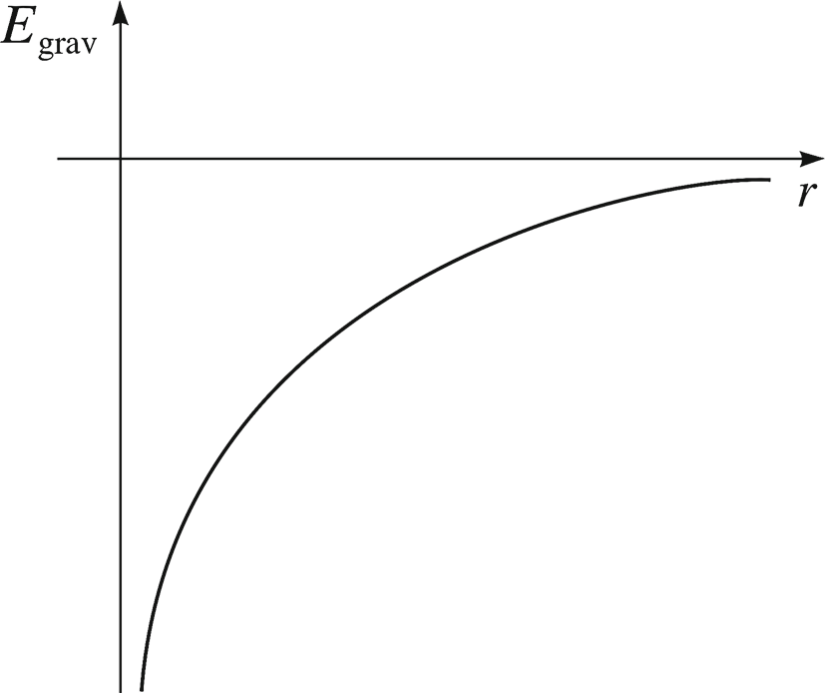
The gravitational potential energy. Here *r* corresponds to ${|q_i-q_j|}$ in Eq. 1

The gravitational potential $V \sim \frac{1}{r}$ is asymptotically zero; and this dominates the behaviour of SGS due to (i) its infinite range and (ii) the fact that a (potentially infinite) amount of energy can be released as two point particles get arbitrarily close together, as seen in Fig. 2.

Here, we primarily focus on the gravitational *n*-body case where stellar systems are treated as collections of *n* point masses. Unlike ideal gases, the total energy is not even approximately the sum of the kinetic energy of the constituents since the (negative) gravitational potential energy must be included. The Hamiltonian for such a system of *n* ‘particles’ of equal mass *m* is thus:

1 $$\begin{aligned} H(\mathbf {q,p})=\sum \limits _{i=1}^n \frac{\mathbf {p_i}^2}{2m}-\frac{1}{2}\sum \limits _{i=1}^n\sum \limits _{i\ne j} \frac{Gm^2}{|\mathbf {q_i}-\mathbf {q_j}|} \end{aligned}$$

Whilst this is an idealisation, it provides a very successful description of elliptical galaxies ($10^{11}$ stars) and globular clusters, i.e. spherical gravitationally bound systems of about $10^5$ stars, which both contain very little interstellar medium (dust and gas).

Of course, for some systems we cannot ignore hydrodynamics—namely when interstellar dust and gas are relevant. And for some systems general relativity cannot be ignored. For example, this applies when black holes are present, and when the cosmological structure i.e. curvature of space on very long length scales, cannot be ignored, such as in the dynamics of clusters of galaxies.

Indeed, I should make an obvious and more general disclaimer: whilst Newtonian thermal physics can be used in galactic dynamics describing extraterrestrial systems it is (unsurprisingly) far from the whole story. Nevertheless, models based on the simple Hamiltonian (1) have had some venerable successes: cf. Sect. 2.2.

### Successes

I shall sketch two approaches, the first assuming stars do not ‘collide’, the second allowing for collisions. To model these gravitating systems, the broad idea is to find a probability density function *f* in phase space and consider its evolution.

Modelling a stellar system to be collisionless requires the approximation that no ‘encounters’ occur. An encounter occurs when two stars are so close as to cause a gravitational perturbation, altering their orbits. (Collisions involving physical contact between stars are exceedingly rare and can be ignored in most models.)

The star’s orbit is then approximated by assuming the total mass of the system is smoothly distributed instead of concentrated in point-like stars. This ‘collisionless’ (encounter-less) approximation holds for certain systems, in particular: for globular clusters and elliptical galaxies (containing about $10^{10}$ stars) since, for timescales less than the relaxation time, stellar encounters are unimportant except at their centres (Binney and Tremaine 1987).

Here, the relaxation time is proportional to the number of stars and the time taken for a star to cross the galaxy (the crossing time). After the relaxation time the star’s actual velocity differs from the smooth gravitational field case and its orbit will deviate from the smooth field model by an amount of the order of its original velocity.

As in Boltzmann’s treatment of a dilute gas, we define a probability density function $f(\overrightarrow{r},\overrightarrow{v},t)$ where $f(\overrightarrow{r},\overrightarrow{v},t) d^3rd^3v$ gives the probability at *t* of finding a star in volume $d^3r$ around *r* with velocity within $d^3v$ of *v*. Since we assume all *N* stars have the same probability density function (and are stochastically independent of each other), this function is defined in a 6-dimensional phase space, rather than the 6*N*-dimensional phase space of the entire set of *N* stars.

The collisionless Boltzmann equation gives this function’s evolution;

2 $$\begin{aligned} \frac{\partial f}{\partial t} +[f,H]=0 \end{aligned}$$

where *H* is given by Eq. 1. Note that the collisionless Boltzmann equation is nonlinear as the gravitational potential $\Phi (x,t)$ depends on the distribution of stars’ masses, $f(\overrightarrow{r},\overrightarrow{v},t)$.

We can define the entropy

3 $$\begin{aligned} S=-N\int f(\mathbf {r},\mathbf {v},t) \ln f(\mathbf {r},\mathbf {v},t)d^3rd^3v. \end{aligned}$$

To look at the evolution of a stellar system over timescales longer than the relaxation time, in which encounters between stars must be considered, we need what is (usually) called the Fokker-Planck approximation. The encounter operator, $\Lambda [f]$, gives the difference of the probability that a star is scattered into and out of a volume of phase space in a given time interval. Equation 2 becomes

4 $$\begin{aligned} \frac{\partial f}{\partial t} +[f,H]= \Lambda [f]. \end{aligned}$$

To sum up: the collisionless Boltzmann and Fokker-Planck equations have proven to be empirically successful evolution equations for the systems described at the end of Sect. 2.1.^1^

### Unusual features

However, the extension of thermal physics to SGS is far from seamless. There are a wide array of problems surveyed in Callender (2011): of which I will consider only three.

**Fig. 3:**
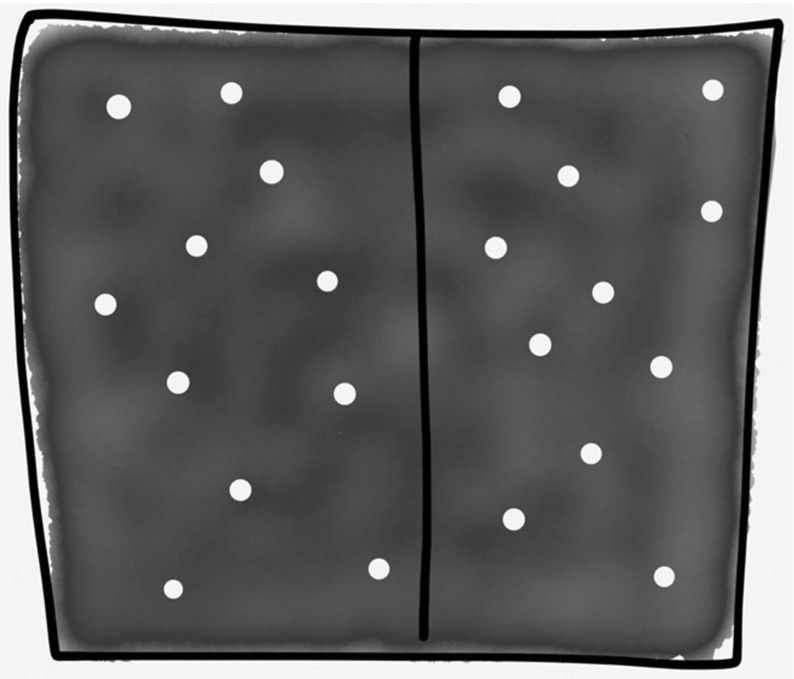
Components interacting via short-range forces, as is the case for an ideal gas at room temperature.

**Fig. 4:**
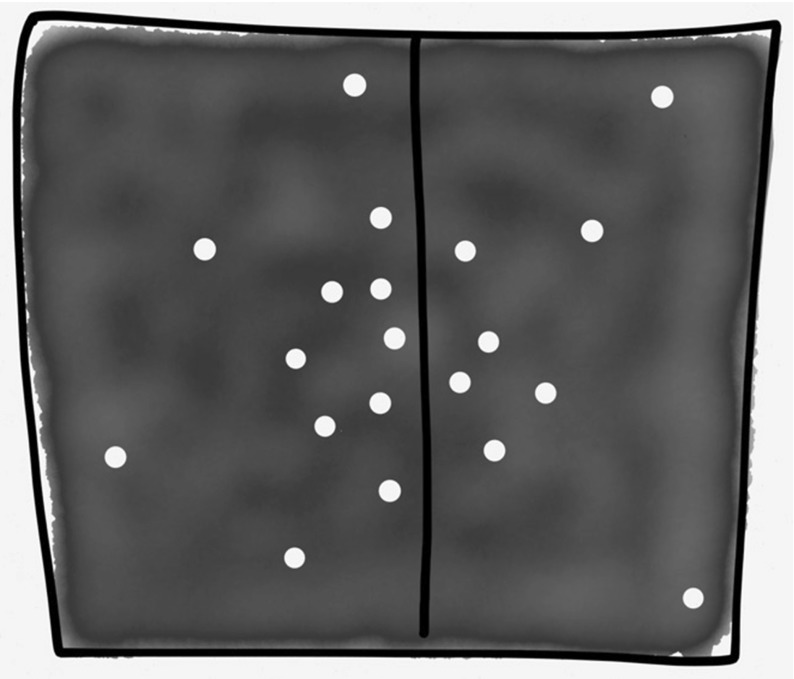
Components interacting via long-range forces, as is the case for self-gravitating systems

(1) *Strong interactions* Firstly, functions, such as energy and entropy, are often not additive or extensive for SGS. For an ideal gas the total energy *E* is the kinetic energy *K*, whereas for gravitating systems the (negative) potential energy *U* contributes: $E=K+U$. Functions such as energy and entropy are usually additive: the energy of a combined system A + B is just the sum of the energy of A and the energy of B. Usually the Hamiltonian of the joint system is $H_{AB}= H_A + H_B + H_{int}$, but it can be approximated by $H_{AB}= H_A + H_B$. (So strictly speaking, the energy is additive iff there are no interactions, i.e. $H_{int}=0$). However, a SGS will not have even *approximately* additive functions since the neighbouring stars do not contribute the majority of the influence on a particular star (Cf. Fig. 1). That is, the interaction Hamiltonian, $H_{int}\not \approx 0$. The physical reason for this can be seen in Figs. 3 and 4, showing how putting together two ‘boxes’ of gravitating stars alters both boxes: the long-range attractive forces result in ‘clustering’ or ‘clumping’ not seen for ideal gases (or indeed real gases in terrestrial settings, which are well described by zero or only short-range forces between constituents). For these gases, short-range potentials are dominant—adding two boxes of gases does not alter the systems in such a dramatic way, since the systems only interact at their boundary.

As a consequence, variables such as energy and entropy are usually taken to be *extensive*. Here, a variable is called ‘extensive’ if it depends linearly on the size of, i.e. the number of constituents in, the system (e.g. mass, internal energy, volume)^2^ and is called ‘intensive’ if independent of system size (e.g. density, pressure). The energy of a subsystem is proportional to the volume, whereas interactions between subsystems are proportional to their interface boundary’s surface area and are, therefore, of a smaller order of magnitude, provided the subsystems are big enough. So strictly speaking, even for short-range potentials, entropy and energy are only extensive in the thermodynamic limit. But although this is a matter of degree, there is still a contrast of principle with SGS. For energy and entropy are not extensive for gravitating systems, no matter how large the system.^3^

(2) *Putting in energy reduces the temperature* Gravitating systems can have a very unusual property: negative heat capacity. The *heat capacity* (at constant volume) is the amount of energy required to raise the temperature by one degree at constant volume;

5 $$\begin{aligned} C_V= \frac{\partial E}{\partial T}\bigg |_{V}. \end{aligned}$$

When the system is in virial equilibrium (where $2K+U=0$), the total energy is negative ($E=K+U$, so $E=-K$, where *K* is by definition positive). From the equipartition theorem , we have $K=\frac{3}{2}Nk_BT$. This implies $E= -\frac{3}{2}Nk_B T$ and thus $C_V=-\frac{3}{2}Nk_B$: the heat capacity is negative. If the system gives out energy, the temperature will increase. If you put energy into a system, the temperature goes down. Indeed, unusual!

(3) *The gravothermal catastrophe* Thirdly, there is the infamous gravothermal catastrophe (Lynden-Bell et al. 1968). To explain this, let us consider in general terms which evolutions are entropically favourable. Whether a process (such as expansion) increases entropy depends on whether the phase space volume increases. Thus, for example, expansion of an ideal gas is entropically favoured since it increases the volume available. Ceteris paribus, the hotter the system the higher its entropy as more momentum states are available (due to the increased kinetic energy). So whether an expansion of a self-gravitating system increases or decreases entropy depends on how the competing factors affect the phase space volume (Wallace 2010). An increased volume means more spatial states but results in a decreased number of momentum states as the kinetic energy has decreased, since work is done against the attractive gravitational field.

Turning now to SGS: when the density contrast between the edge and centre of a SGS is great enough, we conceptually divide the system into a uniform core and a uniform halo, each in virial equilibrium. If a small amount of heat is transferred to the envelope from the core, the core’s kinetic energy decreases, making it favourable for the core to contract (as $U=2E$, *E* has decreased so *U* is more negative). Since the core has negative heat capacity, losing energy increases the temperature. The core decreases in entropy but this is more than offset by the expansion and cooling of the halo.^4^ The heat flow and contraction increases the temperature gradient between the core and envelope and thus the process of heat transfer from the core to the halo is self-perpetuating.

The gravitational potential, $V\sim \frac{1}{r}$, being unbounded from below as $r\rightarrow 0$, means that this collapse would appear to continue without end. For an infinite amount of potential energy can be released by moving two particles closer and closer together, as seen in Fig. 2. Consequently, it seems that there are no equilibrium states. No equilibrium will be reached since, according to the gravitational potential, the core can keep contracting indefinitely becoming infinitely dense.

Is this gravothermal collapse observed? Here we meet a familiar philosophical theme: that singularities in one theory can signify the breakdown of that theory, and often signal some features of the successor theory (Berry 2002; Batterman 2001)—so that idealisations taking some quantity to infinity can play a key role in inter-theory relations. More generally, physics consists of models which have a limited domain of applicability; if you push any model of physics far enough it will break down. As Feynman quips: “When you follow any of our physics too far, you find it always gets into some kind of trouble” (Feynman et al. 1964, §28.1). The same point is made in the literature about SGS: Hut says “whenever a theory predicts the occurrence of singularities, it has been a sign that other physical effects, which have been overlooked, will kick in before actual infinities are reached” (Hut 1997).

But to return the question of gravothermal collapse: indeed, as Hut says, other physical effects eventually kick in. Globular clusters undergo this gravothermal collapse, albeit over a period of tens of millions of years. Agreed: in a globular cluster, the formation of hard binaries provides the core with an energy source (Spitzer and Ostriker 1997, p. 363): nevertheless, once exhausted gravitational collapse will continue. Another instance is a contracting gas cloud (that ultimately will form stars) where the heat is emitted as electromagnetic radiation (due to the presence of an interstellar medium which is absent from globular clusters). In the case of stars, fusion processes provide the energy source to resist gravitational collapse but eventually this energy source runs out. In this case, gravitational collapse resumes until another effect (dependent on the star’s mass) kicks in. For example: for stars of around 10 solar masses, collapse continues until a supernova occurs leaving a neutron star in which the degeneracy pressure (a consequence of the Pauli exclusion principle) resists the attractive force of gravity (Phillips 2013).

But I will not need more details about these “additional physical effects”. For this paper, the main point of all these other effects is that they involve various theories and subdisciplines of physics such as hydrodynamics, quantum theory—and statistical mechanics (cf. Sects. 4 and 5).^5^

## My strategy for reconciliation

Callender (2011) argues that to reconcile the two sides of the debate, we should take a broader, more liberal view of thermodynamics. We should not ‘take thermodynamics too seriously’ but allow for such unusual features. For example, equilibrium needn’t be strict (Callender 2001).

Thus Callender asks: what should we conclude from SGS’s unusual features? He says *“If there is a general lesson, I believe it is that we sometimes have too narrow a vision of thermodynamics. In his beautiful review*, Thompson (1972) writes that ‘to show that thermodynamics exists for a given system’ we must (a) ‘prove. . . the existence of the thermodynamic limit’ and (b) ‘show that the resulting thermodynamics is stable’, i.e., prove that specific heat is positive. By these criteria, self-gravitating systems badly fail as thermodynamic systems. Yet thermodynamic techniques sometimes have proven successful when applied to self-gravitating systems. How do we reconcile these two facts?” (Callender 2011, p. 979).

I advocate a different view: by dividing thermal physics into thermodynamics and statistical mechanics, no reconciliation is required. This is because phenomenological thermodynamics does not apply to these systems (a claim I argue for in Sect. 4), although, to a certain extent, statistical mechanics does (a claim I argue for in Sect. 5). Thus, the dispute over the applicability of thermal physics is deflated as merely semantic: the Optimists are talking about SM whereas the Pessimists are talking about TD.

Of course, dividing thermal physics into TD and SM is an incredibly contentious matter. Can one draw a clean line and if so, where should one draw it? I submit that some division, albeit a rough or vague one, must be possible, as a prerequisite of the meaningfulness of the reduction debate, which after all requires that there are *two* theories, one of which may or may not ‘reduce’ to the other. (And whatever one’s qualms about the reduction debate, to say it is meaningless is surely just intellectual defeatism).

*That* such a line can be drawn is a prerequisite of the reduction debate and *how* it is drawn is important for whether a reduction exists: for claims of reduction are evaluated not only in relation to a given account of reduction, but also in relation to the definitions of the two theories.

I agree that there are multiple possibilities of how to draw the line between TD and SM—and these different options have various foundational motivations. There is a plurality of ways to carve up the terrain, and how one does it depends on one’s aims. Thus if you believe that SM is the powerhouse of thermal physics (Wallace 2015), your preferred line might be different from those who venerate thermodynamics (such as Eddington 1928, p. 104). In addition to this question of the conceptual priority of TD or SM, the foundational debate between Gibbsians and Boltzmannians plays a role. For instance, one might advocate a Gibbsian definition of equilibrium (that the probability distribution is stationary) because it nicely lines up with the thermodynamic definition (that the macrovariables are stationary). This is because the phase average of a macrovariable^6^, using a stationary probability distribution will also be stationary—so with reduction in mind, this Gibbsian definition is a good SM candidate for reducing TD equilibrium. On the other hand, Callender advocates a Boltzmannian view of SM: according to which equilibrium is defined to be the largest macrostate and the system can fluctuate away from equilibrium, which is arguably unlike the traditional thermodynamic definition. In order that reduction is still on the cards, Callender advocates taking a more liberal view of thermodynamics (Callender 2001). Thus, Callender’s reconciliation between the Optimists and the Pessimists is part of his wider view of the foundation of thermal physics. Hence, the line I propose between TD and SM in what follows may have different foundational motivations than that of Callender and others.

Thus, whilst some split must be possible, that is not to say it is either precise or wholly objective. Thermodynamics and statistical mechanics developed ‘cheek by jowl’ and so a sharing, indeed a blurring of concepts, methods and results seems inevitable. As part of the historical progress of science, the original meaning of theoretical terms in one theory bends under the success of another. As is well-documented, the success of SM led to conceptual extensions of TD, such as negative temperatures. Indeed, one might see this as evidence of a successful diachronic reduction, with SM the successor theory. Furthermore, this explains how such a semantic dispute could arise between the Optimists and the Pessimists; often physicists talk of ‘statistical thermodynamics’ and arguably it is the (putative) reduction that has led to the blurring of the two theories for practical purposes.

But the putative reduction of TD to SM is not only a diachronic reduction of an older theory to its successor, but also a synchronic reduction of the higher-level macroscopic theory to the lower-level underlying microscopic theory. The inter-theoretic relationship between TD-SM differs from the classic diachronic reduction between Newtonian mechanics and Special Relativity. Newtonian mechanics is wrong in certain domains and predictions, but it is contested whether thermodynamics is wrong in the same way. This is made especially clear by those who venerate thermodynamics, claiming it to be fundamental. Planck, for example, took the second law of TD to be universal, applicable to “every process occurring in nature” (Planck 1926, p. 463) (as quoted in Uffink 2006, p. 280). One classic exponent of this view is Eddington, who claimed that: *“If someone points out to you that your pet theory of the universe is in disagreement with Maxwell’s equations—then so much the worse for Maxwell’s equations. If it is found to be contradicted by observation—well, these experimentalists do bungle things sometimes. But if your theory is found to be against the second law of thermodynamics I can give you no hope; there is nothing for it but to collapse in deepest humiliation”* (Eddington 1928, p. 104). Such a view is certainly at odds with comparing TD to the (superseded) Newtonian theory when discussing diachronic reduction. Thermodynamics is not straightforwardly ‘inferior’ to statistical mechanics.

But in the case of SGS I will argue: TD does not apply but SM does. And it is of foundational significance *which* theory these successes of thermal physics in this exotic domain belong to. This is because of the above question of the conceptual priority of TD and SM, which influences the reduction debate. For instance, one possible—if not popular—position is that it is a failing of the Boltzmannian entropy that it does not *strictly* increase, whereas it is a virtue of the Gibbs coarse-grained entropy that it does: because this is more faithful to the thermodynamic entropy (cf. Callender 2001). Thus, not only must we have two distinct theories and a definition of each, but we also need to be clear on the conceptual priority of one over the other.

Despite these connections and complications surrounding reduction and the sharing, indeed blurring of concepts, I contend that two core frameworks can be distinguished—though of course, boundary cases may still remain. In broad terms, this goes as follows. TD is an abstract theory, that proceeds in ignorance of the constitution of the system, dealing instead only with macrovariables which obey the Four Laws (or really, Five Laws—cf. Sect. 4 on the “minus first law”). In contrast, SM describes systems by considering statistical, or probabilistic, distributional features of the microvariables. In particular, the state space of equilibrium thermodynamics consists of equilibrium states labelled by a small number of macrovariables, whereas the state space of statistical mechanics consists of appropriate probability density distributions over microvariables, such as position and velocity of the microconstituents. In order that we do not beg the question about reduction, it is important that we keep the concepts of each theory distinct. Accordingly, the concepts of SM, in particular a SM notion of entropy or equilibrium *may* turn out to identical to the thermodynamic entropy or equilibrium—but this would be a major case of theoretical identification and so should not be assumed at the outset.

Having admitted the difficulties with dividing thermal physics into SM and TD, I now offer two reasons why my position—the debate between the Optimists and Pessimists can be deflated, because TD does not apply although (to an extent) SM does—might be anticipated/seem natural.

Firstly: as highlighted in the introduction, TD was created in a time when there was much scepticism about the existence of atoms. Because of this uncertainty surrounding atoms, TD arose as a theory of empirical generalisations about the bulk properties of matter, without regard to its microscopic composition. Einstein famously called thermodynamics a ‘principle theory’, in contrast to those ‘constructive theories’ that ‘build complex phenomena out of relatively simple postulates’ (Einstein 1919, p. 228).^7^ The generalisations of TD were extrapolated from regularities in phenomena familiar from tabletop systems of gases, pistons etc. Thus it is unsurprising that these generalisations do not hold in the radically different realm of stars and galaxies. But the constructive theory now considered to underpin the generalisations of TD—SM—may well apply (and this is considered in Sect. 5).

Secondly: my view is already suggested by some of the physics material reviewed above. The thermal physics of SGS never abstracts away to macroscopic bulk variables from the microvariables—i.e. the position and momenta of the individual stars—and probability distributions over these microvariables.^8^ And indeed, Sect. 2.3’s discussion of the gravothermal catastrophe used statistical mechanical notions of entropy. Furthermore, the collisionless Boltzmann equation and the Fokker-Planck equation for SGS originate from *non-equilibrium SM*...while it is equilibrium SM to which TD putatively reduces. So the inapplicability of TD should be anticipated.

In the next section, I develop the sketch above, of what I take to be the key features of thermodynamics, and then argue that the thermal physics used in SGS cannot be thermodynamics.

## Thermodynamics “Construed”

I will first present my perspective on thermodynamics in general (Sect. 4.1), and then argue that thermodynamics so construed, does not apply to SGS (Sect. 4.2).

### Thermodynamics in general

The state space of thermodynamics is the space of equilibrium states, parametrised by two or more macrovariables. For example, for a gas, the points of the state space can be labelled by the pressure and volume (*p*, *V*); for a film, they are labelled by surface tension and area; for a magnet, magnetic field and magnetization; and for a dielectric, electric field and polarization (e.g. Tong 2012, §4).

Thermodynamic equilibrium states—whatever their relation to SM equilibrium states and however idealised—are states in which the macrovariables no longer vary in time: the system (as described by thermodynamics) will sit there indefinitely.^9^ That systems will reach such an equilibrium state has been dubbed the ‘minus first law’ of thermodynamics (Brown and Uffink 2001). Once the system has reached an equilibrium state, its thermodynamic entropy—which is a function of these labelling macrovariables—cannot increase further under *spontaneous* processes. There are no spontaneous trajectories through the thermodynamic state space of equilibrium states: if a system is in an equilibrium state then by definition it will not change. Instead, the state of the system will only change when we perform certain interventions on it, e.g.: inserting a partition, squeezing with a piston, placing the system in thermal contact with another, or with a heat bath... etc. These interventions implement certain thermodynamic processes, such as isothermal compression and adiabatic expansion, by changing external parameters such as volume. For this reason, thermodynamics has been described as a control theory (Wallace 2014). A number of actions can be performed on the controlled system and only certain transitions between states can be induced.^10^ That thermodynamics can be described as a control theory sets it apart from other physical dynamical theories which describe the space of possible states of a system and the system’s *spontaneous* trajectory through that space, which is represented by a curve in that space.

In contrast, curves through the equilibrium state space of thermodynamics—understood as ‘thermodynamic processes’—have been the source of interpretative controversy. As emphasised by Norton (2016) the term ‘equilibrium process’ is oxymoronic: if equilibrium is understood to mean ‘a state in which nothing changes’ then by definition it contradicts a ‘process’—whose meaning is that something changes; cf. also Lavis (2017) and Valente (2018).

For this reason, Tatiana Ehrenfest-Afanassjewa called curves in thermodynamic equilibrium space ‘quasi processes’ to indicate that they were not physical processes at all (Ehrenfest-Afanassjewa 1925, 1956). Instead the system is ‘nudged’ from equilibrium (and so out of the equilibrium state space, cf. Norton 2016, p. 45) by altering one of the control variables/external parameters—e.g. by raising the temperature by putting the system (at temperature $T_1$) in contact with a heat bath at $T_1+\Delta T$. According to the minus first law of TD, the system will reach a new equilibrium state at this new temperature. The process is then iterated with a series of heat baths at different temperatures, and in this sense the system is pushed along the curve.^11^

This picture of curves in equilibrium space involving nudging the system and it returning to (a perhaps new) equilibrium state requires that the system is thermodynamically stable. In particular, it requires that the second derivative of the entropy is negative^12^: $\frac{\partial ^2S}{\partial E^2}< 0$. In terms of other variables, an alternative requirement for stability is that the heat capacity $C_v$ is positive (cf. Landau and Lifshitz 1969, p. 47; Thompson 1972, p. 72).

This is why positive heat capacity is often taken as a principle of thermodynamics. Indeed, as I mentioned in Sect. 2.3 (2): strange non-thermodynamic behaviour can occur with a system with negative heat capacity. For example, if a heat bath B (with positive heat capacity, $C_{v}^{B}$) has a lower temperature than a system S with negative heat capacity $C_{v}^{S}$, heat flows from S to B raising the temperature of both. If $|C_{v}^{B}|<|C_{v}^{S}|$, an equilibrium can be reached where both systems are at a higher temperature than initially.^13^

To sum up: the state space of thermodynamics consists of equilibrium states labelled by a small number of macrovariables. In order for a system to undergo any change, the control variables must be altered by an external system (Lavis 2017). Thus, a curve through this equilibrium state space does not represent any spontaneous process. Instead, a substantive idealisation is in play: and for this to be connected to the behaviour of real systems the system must be thermodynamically stable so that after small changes in the control variables the system returns to another equilibrium state.

### Thermodynamics does not apply to SGS

I claim: the theory discussed above is a far cry from the type of thermal physics used in galactic dynamics, in trying to deal with the ‘gravitational million body problem’. In this section, I will argue in a two-pronged attack that thermodynamics—as construed above—does not apply to SGS.

As the state space of TD is the space of possible equilibrium states, we must first consider: what would count as a thermodynamic equilibrium state for a SGS? It is unclear. Binney and Tremaine simply deny that they are any (Binney and Tremaine 1987, p. 269). Callender is more flexible, suggesting that some unusual states do the job. (However, these unusual candidates are *statistical mechanical* states and so I delay discussion of them until Sect. 5).

The gravothermal catastrophe hints at why: it seems that no equilibrium will be reached since, according to the gravitational potential, the core can keep contracting indefinitely becoming infinitely dense.^14^ At this point we face two options.

Either we maintain that such singularities are unphysical, as discussed in Sect. 2.3. They hint at the breakdown of the theory, and any attendant approximations we may have used to deduce the singularity. No such infinities will be reached because other effects will kick in. In the case of globular clusters, the formation of binary stars provides an energy source to resist gravitational collapse. The question is then at which point is the system in equilibrium, which macrovariables are stationary and over which timescales. And there appears to be no clear answer to this. Thus, “the claim that self-gravitating systems have no equilibrium, in particular, is the norm rather than the exception” (Callender 2011, p. 962) and “galaxies are not in thermodynamic equilibrium” (Binney and Tremaine 1987, p. 571).

The second option is that the collapsed state of infinite density *is* an equilibrium state—after all, nothing will happen. But I submit that even if we dub this state a thermodynamic equilibrium state, it is only *one* state and to do thermodynamics, we need a whole state space of different possible equilibrium states so that, for instance, we can define curves through this space and so discuss adiabatic and isothermal processes (cf. Sect. 4.1). If we have one lone equilibrium state, then we cannot talk of changing an external parameter such as volume in order to ‘nudge’ the system to a new equilibrium state, if there is only one state in the whole state space!

This brings me to the second prong of my attack. Even if we could construct an equilibrium state space, there is another problem: SGS are unstable. Perturbing (‘poking’) the systems, even very gently in the manner required for a quasi static process, can lead to runaway instability. This is unsurprising: even without considering the concavity of entropy (i.e. the condition $\frac{\partial ^2S}{\partial E^2}< 0$) and other mathematical conditions (cf. footnote 12), we know this is *exactly* what happens in gravitational systems—recall the ‘gravitational clumping’ in Fig. 4! Not only will the system be inhomogeneous with respect to the position of the constituents, but the negative heat capacity implies that an initial temperature gradient is exacerbated. If one system loses energy to the other its temperature increases, whilst the system gaining energy has a decreasing temperature, perpetuating the heat flow between them indefinitely, as seen in Sect. 2.3’s gravothermal catastrophe. Initial temperature gradients are accentuated by the heat flow rather than dissipated. This is characteristic of SGS; small inhomogeneities (in the distribution of matter as well as temperature) get amplified not dissipated.

This lack of thermal stability means that after the small interventions used in enacting a ‘thermodynamic process’, the system will not return to a new (and nearby) equilibrium as required in TD, i.e. by the minus first law of TD. Instead, because SGS do not fulfil the conditions discussed in the previous section (such as positive heat capacity) for thermal stability, a small perturbation will lead to a large change in the state of the system.

Finally, as an aside, notice that the perspective of TD as a control theory brings to light the unthermodynamic nature of SGS. The point here is not merely the obvious, albeit amusing, thought that we cannot manipulate a star, let alone a globular cluster or galaxy. (Cf. the quote from Rowlinson 1993 which I earlier took as emblematic of the Pessimists.) Whilst Elson says ‘globular clusters provide an ideal laboratory’ (Elson et al. 1987, p. 565), there are important differences between SGS and ordinary TD system that influence how we “manipulate” these systems in computer simulations.^15^ In particular, different parameters (such as temperature, density, size of the system) cannot vary independently. The volume of the system is determined by the gravitational potential, and thus volume is not independent of the energy of the system. Hut (1997), p. 10 describes a SGS as having only ‘a single coupling constant’—the number of stars. Therefore, even if we could induce the SGS to transition from one state to another, there is only one control variable. As Hut (1997) vividly describes, in a star cluster there are no cylinders or pistons. Instead the stars are confined by their collective gravitational field.

To sum up the argument of this section: I have argued for The Main Verdict: There is no appropriate equilibrium state space for a SGS. Furthermore, the instability of SGS means that the minus first law of thermodynamics does not apply, and consequently there can be no ‘thermodynamic processes’.

## Does statistical mechanics apply to SGS?

I say Yes. That is, the success of thermal physics in application to SGS—such as the collisionless Boltzmann equation etc.—should be attributed to statistical mechanics, not to thermodynamics.

A critic might object that only the mathematical machinery of SM succeeds: stellar systems contain vast numbers of stars (and we can’t even solve the 3-body problem!) and thus the calculational problems we faced for a mechanical description of a gas of $10^{23}$ molecules arise again in the context of self-gravitating systems and so similar mathematical techniques are required. (Indeed, with the good comes the bad: similarities in mathematical success are also followed by similarities in mathematical difficulties. For instance, the scope of SM of SGS is limited to collisionless or weakly interacting systems: some SGS have interactions that are too strong for SM to handle—just like in the case of terrestrial SM! Cf. Callender 2010, §5).

Accordingly, in this section I discuss the extent to which SM applies. I will agree with the above critic: the applicability of SM does have limitations: in particular, the evolution of self-gravitating systems never reaches a SM equilibrium. Nonetheless, the success of SM is not merely mathematical: when a gravitational kinetic equation can be given, the entropy cannot decrease. Thus, it is not merely the mathematical machinery of SM that applies, although the success of SM must be qualified.

### An approach to equilibrium?

Describing *quantitatively* the approach to equilibrium is a key part of the enterprise of SM, known as non-equilibrium SM.^16^ Can we describe the behaviour of stellar systems as an approach to equilibrium? If so, this would be a fundamentally statistical-mechanical explanation of the phenomena.

But indeed, there is a problem. Thus Binney and Tremaine say that “we can always increase the entropy of a self-gravitating system of point masses at fixed total mass and energy by increasing the system’s degree of central concentration” (Binney and Tremaine 1987, p. 268). Consequently, no density function $f(\overrightarrow{r},\overrightarrow{v},t)$ maximises entropy for finite mass and energy.^17^ So if SM equilibrium is taken to be defined as the maximum entropy state (a feature common to both the Gibbsian and Boltzmannian definitions of equilibrium) a SM equilibrium cannot be found for finite systems.

There are three possible reactions. First, Binney and Tremaine conclude that the behaviour of SGS cannot be treated as a relaxation to equilibrium. $f(\overrightarrow{r},\overrightarrow{v},t)$ is not analogous to the velocity distribution of an ideal gas relaxing to the Maxwell-Boltzmann distribution. Galaxies and other typical stellar configurations are not the result of a long-term thermal equilibrium (Binney and Tremaine 1987, p. 269).

Second, Callender (2011) takes a different view and suggests that the unconventional states such as the collapsing core-halo states and similar Dirac $\delta$-function ‘singular peaks’ should “be regarded as equilibrium states for the same reasons cups of coffee at room temperature can be” (Callender 2011, p. 968). If these states can be interpreted as SM equilibrium states, then (on this view) the behaviour of SGS could be an approach to equilibrium.

However, a cup of coffee is in a *local* equilibrium state. Rather than being described by a global Maxwellian distribution such as,

7 $$\begin{aligned} f(p,q)=Ne^{\frac{p^2}{2mkT}}, \end{aligned}$$

the system is in a local Maxwellian distribution. For instance, the temperature of the coffee varies with position, but locally looks like an equilibrium state. Thus over certain distance scales, the coffee looks like it is in equilibrium.

8 $$\begin{aligned} f(p,q)=N(q)e^{\frac{p^2}{2mkT(q)}} \end{aligned}$$

Whilst the cores of stars are in local but not global equilibrium, the unconventional states that Callender proposes are not states of local equilibrium.^18^ Further, by Callender’s own lights the ‘Dirac peak’ is the wrong state to use; since he claims that the canonical ensemble (in which the state is defined) is the wrong ensemble to use for astrophysical systems and instead the microcanonical ensemble should be used (Callender 2011, p. 967).

Thirdly, you can walk straight in a particular direction without reaching some prescribed destination. That is: when a gravitational kinetic equation is given, the entropy (as a function of the distribution function) increases but never reaches a maximum. Were there a maximum entropy state, we might want to call this the SM equilibrium state. For SGS there is no such destination, but nonetheless these systems head in that direction: so I conclude that there is (a weak sense) in which they *approach* equilibrium, although they do not reach it.

This meshes nicely with our earlier discussion in Sect. 4.2. There we saw (in the ‘second prong of attack’) that due to the instability of SGS, the minus first law of TD does not hold: SGS do not spontaneously return to TD equilibrium after small perturbations. I cannot of course go to into detail here about the exact relations between SM equilibrium and TD equilibrium. But we expect non-equilibrium SM to in some way justify or underpin the minus first law of TD. So not reaching SM equilibrium (and consequently limiting the applicability of SM) fits with my earlier conclusion that the minus first law does not apply to SGS. Furthermore, as we saw in the ‘first prong of attack’, SGS do not reach a state of thermodynamic equilibrium. Had SM equilibrium states been available this may have suggested that there is a way to find a TD equilibrium state space, since SM equilibrium is meant to (in some sense) ground TD equilibrium. Thus, the lack of SM equilibrium further supports the conclusion of Sect. 4.

### Surprise?

Should we think it surprising that statistical mechanics applies here? I say: No. The application of SM to SGS is not surprising. For very similar assumptions are used in the descriptions of dilute gases and of the SGS that SM is capable of describing. First, the collisionless Boltzmann equation assumes that the stars in the system are intrinsically identical (in particular having the same mass m) and non-interacting—just as Boltzmann assumed for an ideal gas. Secondly, an assumption similar to the Boltzmann’s infamous ‘Stosszahlansatz’ is made: the presence of star 1 being found in a particular area of phase space does not raise or lower the probability of star 2 being found there (Binney and Tremaine 1987).

The application of SM may not be surprising, but it might nonetheless still be surprising that we seem to have an SM description *without* a TD description. After all, the concepts of each theory are frequently assumed to be intertwined: as discussed in Sect. 3, the two theories are not always cleanly separated. But in the next section I given an explanation of why no TD behaviour emerges from the SM description.

## The bottom up explanation

I have concluded that whilst there is (to some extent) a SM description of SGS, thermodynamic behaviour does not emerge. This conclusion allows a peace to be made between the Pessimists and the Optimists. The Pessimists were sceptical that theory concerned with steam engines could be extrapolated so far from its human origins to such exotic realms. To the extent that they are talking about the applicability of *thermodynamics*, they are correct. But the Optimists are correct too—thermal physics *is* successful in these exotic gravitational realms—provided that by thermal physics we mean *statistical mechanics*.

I finish by sketching an explanation of this state of affairs: SGS can be given a SM description, but thermodynamic behaviour does not emerge. The explanation of this fact is that a particular mathematical limit—the thermodynamic limit—does not exist (Padmanabhan 1990, p. 295). The topic of the thermodynamic limit is a vast one (see Ruelle 1999 for a classic presentation of both continuous and discrete systems). The key question is whether there is a mathematically well-defined ideal infinite system obtained by $n\rightarrow \infty$, where *n* is the number of constituents of a system.^19^ Usually, the thermodynamic limit not only takes $n\rightarrow \infty$ but also fixes the density, i.e. $\frac{n}{V}\rightarrow k$, whilst $n\rightarrow \infty$.

*The Bottom Up Explanation:* Generically, in the thermodynamic limit, thermodynamic behaviour emerges from the SM description. But the thermodynamic limit does not exist for self-gravitating systems.

In filling out this explanation, I will discuss: (1) why the limit does not exist and (2) the significance of its not existing.

*(1) The thermodynamic limit does not exist for self-gravitating systems.* To prove the existence of a thermodynamic limit, it suffices to show that two conditions are met by the system under consideration (Penrose 1979, p. 1963), see also (Thompson 1972, ch. 3) and (Ruelle 1999, ch. 3):

1. *Tempering* the interaction between distant particles must be negligible. Here is an example of a system satisfying this tempering condition: a pair potential *U*(*x*) (where *x* is the distance between the two particles) has a finite range if there is a distance $R_0$ such that $U(x)=0$ for $|x|\geqslant R_0$.
2. *Stability* the interaction is stable: if there is a real number $$B\geqslant 0$$ such that the potential energy of *n* particles located at $$x_1,...x_n$$, $$\forall x\forall n$$$$U(x_1, x_2...x_n) \geqslant -nB$$.

These two conditions are violated by the long-range and short-range nature of the gravitational potential respectively. 1. *Tempering* is violated because the gravitational potential between two distant particles (i.e. stars) is $V \sim \frac{1}{r}$, which is unlike the potential above: whilst the potential decreases with distance, $U(x) \ne 0$ for any |*x*|. As we saw earlier, the interaction between a star and its distant neighbours is not negligible, but indeed quite the reverse: as seen in Fig. 1, the long-range nature of the gravitational potential dominates the behaviour of the cluster. Of course what counts as ‘negligible’ is not categorical^20^, but for no degree of accuracy does it seem we can treat these gravitational interactions as ‘negligible’. 2. *Stability* has two components. Firstly, the potential energy must be bounded from below; condition 2 states that there must be a number, $-nB$, such that the potential energy is always greater than this number. But, as seen in Fig. 2 the gravitational potential is not bounded from below: as $r\rightarrow 0$, $U\rightarrow -\infty$. Secondly, *Stability* requires that *U* does not grow faster, as a function of *n*, than *n*. This too is violated by the gravitational potential. Hut (1997), p. 8 calls the gravitational potential ‘superextensive’: $U \sim n^{\frac{5}{3}}$.^21^

Thus, SGS differ from ordinary thermodynamic systems in (at least) two respects: the thermodynamic limit does not exist and energy is not extensive for SGS.^22^

*(2) What is the significance of the lack of limit?*

In full generality, this is a hard question to answer. But here our task is smaller: to explain why an SM description of SGS is successful, but yet no TD behaviour emerges. Given how the two theories are seemingly interwoven, how do we have the applicability of one without the other? The answer is that the thermodynamic limit connects the two theories, but because the limit does not exist for SGS, we should be less surprised at the applicability of one without the other.

I shall briefly spell out three examples of the thermodynamic limit connecting SM and TD. Here the idea is that the differences between the TD description and the SM description are washed out in this limit:^23^

(A) The thermodynamic limit is usually used to reveal features of SM functions. For instance, the canonical and microcanonical ensembles are equivalent in the thermodynamic limit. The significance of this result is sometimes glossed as: the equivalence of ensembles in the limit shows that the same thermodynamic functions (and so behaviour) results—no matter which ensemble is used to calculate those functions.

(B) SM descriptions involve probabilities, whereas the TD descriptions are categorical. But in the thermodynamic limit, the probability of fluctuations away from the mean value (e.g. the mean energy), tend to zero. Thus, in the limit, the SM description becomes more akin to the categorical TD description.

(C) Furthermore, only in the thermodynamic limit are certain SM quantities extensive. For instance, the Gibbs free energy is only extensive in this limit. Or, as seen in Sect. 2.3, $H_{12}=H_1+H_2$ is only an approximation, even for short-range potentials present in familiar cases. Yet, the energy of a subsystem is proportional to the volume, whereas interactions between subsystems are proportional to the boundary’s surface area—and so scaling means that interactions thus become negligible, provided the subsystems are big enough. Thus strictly speaking, even for short-range potentials, entropy and energy are only extensive in the thermodynamic limit.

Because some SM quantities are only truly extensive in the thermodynamic limit, they are only identical to their TD correlates in this limit. Thus, it is usual to say that in the thermodynamic limit, the thermodynamic formalism/functions are recovered from the SM description. For example, Oliver Penrose claims that “the first objective of the study of the thermodynamic limit is to demonstrate that in this limit, the laws of thermodynamics apply and to justify our statistical mechanical recipes for calculating thermodynamic functions...” (Penrose 1979, p. 1957).

But is the thermodynamic limit a necessary and/or a sufficient condition for recovering TD behaviour from a SM description? (Callender 2011, p. 975) suggests it is neither necessary nor sufficient. I agree that it is not established to be a *necessary* condition: there *could* exist a type of system for which the thermodynamic limit of a SM description does not exist but TD nonetheless applies.^24^ Indeed, the role of the thermodynamic limit is not the blanket prescription that one must prove the existence of the thermodynamic limit, in order to be licensed to use the laws of thermodynamics. Instead, we apply the ideas of thermodynamics to a system just when it is useful to do so. This leaves open the possibility that thermodynamics might useful for a system for which the limit does not exist.^25^ But I contend, contra Callender, that SGS do not give us this counterexample, since—as I hope to have established in Sect. 4.2—TD is inapplicable to SGS.^26^

To sum up: the fact that the thermodynamic limit does not exist for SGS explains why the applicability of SM and TD come apart for these systems. Leaving aside SGS, we generically get back TD from SM in the thermodynamic limit. Whether such a state of affairs counts as a case of inter-theoretic reduction will depend on one’s account of reduction (cf. Sklar 1995, ch. 9). In the next subsection I briefly highlight two connections between the debate at hand and the reduction debate. Then I link the main focus of this paper—the domain of applicability of thermodynamics—to the reduction debate.

### The connection to reduction 

The first connection is as follows: there is a debate about whether the role of the thermodynamic limit in SM descriptions of phase transitions blocks the reduction of TD to SM. Briefly, the concern is that a singularity in the free energy can only be achieved in the infinite limit^27^ and this infinite idealisation is unrepresentative of real systems and so some contend that a complete reduction to SM is unavailable. Others argue that the use of the limit need not block reduction and the relevant ‘singular type’ behaviour is seen ‘on the way’ to the limit (Butterfield 2011). Does the above discussion reveal that the thermodynamics limit is crucial (in a way problematic for reduction) even away from the contentious case of phase transitions? Should it be worrisome that functions such has the Gibbs free energy are only extensive (and thus like their TD counterparts) in the thermodynamic limit? I think not. Rather than a qualitative difference that springs out only at the limit—causing water to boil and other phase transitions to occur—nothing so glamorous happens. Rather, here the situation is one of ‘mathematical tidiness’, i.e. the interaction Hamiltonian *really* is $= 0$, rather than $\approx 0$ . Indeed, I contend that if reduction were thwarted by the ‘exactness’ only existing in the ‘unrepresentative’ limit, then this would set the bar so high for inter-theoretic reduction that few or no cases would pass it.

The second connection is a more serious worry for reduction raised by Batterman (2010): he explicates Gibbs’ reticence to talk about thermodynamic identities, who instead discusses only ‘analogies’. The concern is that the plurality of Gibbsian ensembles leads to a plurality of microphysical correlates for the thermodynamic entropy.^28^ Which is the correct one? In the thermodynamic limit the ensembles are equivalent and so Batterman contends that this worry disappears. Thus, the thermodynamic limit plays an important role in discussions about reduction here too.

I now connect the question of domain of applicability of thermodynamics to the reduction debate. Had TD applied to SGS, this could have been used to support the view that ‘thermodynamics is fundamental’, in the manner of Eddington’s and Planck’s views (cf. Sect. 3). But instead, this case study arguably adds to the conceptual priority of SM. That SM applies without the emergence of TD behaviour agrees with the moral of Wallace (2015); SM is foundationally important *not only* insofar as it is connected to TD.

Of course, the more charitable interpretation of the ‘TD is fundamental’ view is that it is merely stressing the importance of TD: in particular, one might read the Eddington quotation in Sect. 3 as emphasising the epistemic security of TD. The claim is that the principle of entropy increase is a principle for which we have vast amounts of evidence; in part because the domain of applicability of thermodynamics is taken to include everyday occurrences such as people ageing, buildings crumblings and coffee cooling. (Such a universal scope is also part of Albert and Loewer’s ‘Mentaculus’ project, cf. Albert 2000; Loewer 2018). But the case of SGS heeds us to be cautious: the scope of TD is not universal.

## Conclusion

The detailed empirical success of our descriptions of SGS form a fascinating, often stunning, part of physics. But it is a success to be credited to the framework of ideas provided by statistical mechanics, not thermodynamics. Thus, the situation is: there is a SM description of SGS such as globular clusters and elliptical galaxies, but no thermodynamic behaviour emerges. The unusual unstable behaviour of SGS, negative heat capacities and runaway instabilities, is alien to thermodynamics—but this is unsurprising when we consider the principle theory of thermodynamics as a control theory whose state space is that of equilibrium states. In contrast, the constructive theory of SM applies to SGS; we can write down a probability distribution for a given star to occupy a certain position and have a certain velocity and that entropy associated to that distribution is non-decreasing. The applicability of SM without the emergence of TD behaviour has a bottom up explanation: the thermodynamic limit does not exist for SGS and so this infinite limit provides a key insight into the connection between SM and TD.

## Footnotes

1 For some examples of solutions to the collisionless Boltzmann equation, the initial conditions and approximations involved, see (Heggie and Hut 2003, ch. 8). Much of the research in this area focuses computational simulations (cf. Sect. 4.2). One useful class of models that take the velocity distribution to be isotropic is Plummer’s model, named after Plummer who used this approximation to fit the observed light distributions of clusters (Spitzer 1987, p. 13).

2 More generally, an extensive variable *Q* is homogeneous (in the modes $n_c$) iff $Q(kn_1,kn_2,..)=kQ(n_1,n_2,...)$.

3 Callender (2011, p. 974) takes the failure of extensivity to be a key problem, but suggests that perhaps extensivity can be recovered through the Kac prescription (a rescaling of the temporal and spatial parameters such that the constant $c:=-Gm^2$ in the Hamiltonian (1) becomes $c=\pm \frac{1}{N}$). Since the merits of this prescription is an open issue in physics, I do not explore it further here.

4 Conservation of energy requires that the heat flow from the core to the halo increases the halo’s energy—which is now *less* negative. Thus, it is favourable for the halo to expand and cool.

5 Callender attempts to abstract away from these details above by altering the gravitational potential by introducing a short cut-off potential $\eta$ which prevents the gravitational potential $\rightarrow -\infty$ as $|q_i-q_j|\rightarrow 0$ as the potential is now bounded from below.

6 $$\begin{aligned} \frac{1}{|{\mathbf {q_i}-\mathbf {q_j}|} \rightarrow \frac{1}{\sqrt{(\mathbf {q_i}-\mathbf {q_j}})^2+\eta ^2}} \end{aligned}$$

Perhaps this short distance cut-off is artificial, but regardless of whether we impose it, there are still no equilibrium states in the sense of a state with maximum entropy.

6 Of course, why Gibbs phase averaging works is a source of controversy, cf., e.g. Malament and Zabell (1980).

7 This distinction, though announced in a ‘mere’ newspaper article has had a great legacy, e.g. in the debate about the primacy of matter vs. geometry in the philosophy of spacetime, e.g. Brown (2005) and Janssen (2009).

8 Interestingly, away from the Newtonian gravitation regime, matters may be different: the no-hair theorems show that a black hole can be characterised by a few bulk variables: its mass, angular momentum and electric charge.

9 Of course, *absolute* equilibrium is a fiction: it is not realised exactly by any system in the world. Insofar as the systems in question are also described by classical mechanics, then—due to the Poincaré recurrence theorem—we know that given enough time any system will return arbitrarily close to its initial state and thus not remain *indefinitely* in a given state. Thus, Feynman is meant to have quipped that ‘equilibrium is the state the system gets into after the very fast stuff [e.g. transients] is over, but the very slow stuff [e.g. Poincaré recurrence] has yet to begin’. But the key point is that we get away with treating a system *as if* they were in thermodynamic, i.e. absolute, equilibrium, because the ‘very fast stuff’ is over and the ‘very slow stuff’ does not matter for the phenomena we are interested in.

10 Sometimes thermodynamics is also referred to as a resource theory, or as means-relative, e.g. Horodecki and Oppenheim (2013).

11 Whether this is an approximation in the sense of Norton (2012) (and whether it can be done reversibly etc.) is beyond the scope of this paper.

12 Here are some details. To check that the system is not unstable with respect to spontaneously becoming inhomogeneous, (Avoras 2013, §2.10) imagines splitting the system—already in equilibrium—into two uneven halves (on the left $(E+\Delta E, V+\Delta V, N+\Delta N)$ and on the right $(E-\Delta E, V-\Delta V, N-\Delta N)$ and then he asks: will the entropy increase or decrease? Using $\Delta S = S(E+\Delta E, V+\Delta V, N+\Delta N)+ S(E-\Delta E, V-\Delta V, N-\Delta N)-S(E, V, N)$, he shows that in order that $\Delta S =0$, the entropy must be a concave function of (*E*, *V*) at fixed *N*.

13 If the converse is true, $|C_{v}^{B}|$>$|C_{v}^{S}|$, then the heat bath B will not increase its temperature ‘quickly enough’ as energy flows in. That is, the system’s temperature will increase faster than the heat bath’s temperature and so they will not reach the same temperature.

14 “Doing nothing, whilst perhaps difficult for human beings, is altogether excluded for a self-gravitating star system” (Heggie and Hut 2003, p. 45).

15 For an insight into the difficulties—beyond the sheer size of *N*—with such computational models, see (Heggie 2003, p. 83).

16 Thermodynamics only states that a system will go to equilibrium (a tendency that has been dubbed the “minus first law” of thermodynamics, as noted in Sect. 4.1). It does not say anything about how fast equilibration occurs.

17 A density function that maximises entropy exists only for the isothermal sphere which has infinite mass and energy (Binney and Tremaine 1987, p. 268).

18 Individual stars are an interesting case: are they examples of SGS that admit of a TD description (and so provide a counterexample to my thesis)? Since they are in local equilibrium, perhaps some of the problems I raised in Sect. 4.2 for SGS such as globular clusters (i.e. ‘no equilibrium states’) do not apply. Yet the equilibrium state in Eq. 8 is a probability distribution over microvariables *p* and *q*—which, according to my classification of Sect. 3, is an example of SM, rather than TD, machinery. What to conclude? I think it will depend on how one draws the line, i.e. what physics one designates as ‘thermodynamics’. Thus, at the outset of his classic monograph ‘Thermodynamics of the Stars’, E.A. Milne distinguishes two senses of thermodynamics: (i) a ‘restricted sense as denoting the study of the equilibrium states of enclosed systems’ and (ii) a ‘general way to denote the study of all those phenomena in which temperature plays a part... the science of heat transfer’ (Milne 1930, p. 5). A more permissive definition of TD—along the lines of Milne’s (ii)—may classify this case as TD. But for my account propounded in this paper, individual stars seem to be a boundary case—and one deserving of further study.

19 I should note that how such procedures should be understood and classified is the topic of philosophical debate, e.g. Norton (2012).

20 I am grateful to an anonymous referee for pressing this point.

21 The ‘back of the envelope’ justification Hut gives for this: “Take a large box containing a homogeneous swarm of stars. Now enlarge the box, keeping the density and temperature of the star distribution constant. The total mass *M* of the stars will then scale with the size *R* of the box as $M\propto R^3$, and the total kinetic energy $E_{kin}$ will simply scale with the mass: $E_{kin}\propto M$. The total potential energy $E_{pot}$, however, will grow faster: $E_{pot}\propto \frac{M^2}{R}\propto M^{\frac{5}{3}}$. Unlike intensive thermodynamic variables that stay constant when we enlarge the system, and unlike extensive variables that grow linearly with the mass of the system, $E_{pot}$ is a superextensive variable, growing faster than linear” (Hut 1997, p. 8).

22 Interestingly, despite their surface similarities, the gravitational potential and Coulomb potential differ: the thermodynamic limit has been proven to exist for certain electromagnetic systems (cf. Lieb and Lebowitz 1972; Thompson 1972, p. 71). But this proof requires quantum considerations (roughly, that there is a ground state so that the energy is bounded from below, so that the stability condition is satisfied). The tempering condition can also be satisfied by electromagnetic systems. The presence of both positive and negative charges leads to Deybe shielding (Callender 2011, p. 961): the force due to distant charges is ‘shielded’ so $U(x)=0$ for suitably large |*x*|. But there is no analogous effect for Deybe shielding for gravitational systems because the gravitational force does not ‘saturate’ (Lévy-Leblond 1969).

23 But of course, there is much more work to be done to understand how the limit connects SM and TD; here I only give a suggestive sketch.

24 Of course, it would be an interesting development if the existence of the thermodynamic limit was a necessary condition for the applicability of thermodynamics —but this is not something I can establish here. I also leave the question of whether the thermodynamic limit is a sufficient condition to one side. If it is a sufficient condition for the applicability of TD, this helps rule which systems fall under the purview of TD. But it would be less enlightening for ruling systems *out*—after all, there could exist an alternative sufficient condition for the applicability of TD. Furthermore, analysing the counterfactual claim that ‘had the TDL existed, then TD would have applied’ is hard for SGS: for the TD limit to exist, the system would have been fundamentally different—as I argued in Sect. 2, the form of the gravitational potential dominates the behaviour of SGS. And it is this potential which fails the tempering and stability conditions.

25 Black holes are a putative example—but much controversy surrounds this claim.

26 Whilst it is conceivable that there is a system for which the thermodynamic limit does not exist and yet thermodynamics is useful, there are reasons to be confident of the importance of ‘large N’ in what is after all, a science of the *bulk* properties of matter. For instance, according to the Boltzmannian perspective on SM, systems inevitably approach equilibrium because of the overwhelming vastness of the equilibrium macrostate compared to the other macrostates. But this requires N to be large—otherwise there will not be one macrostate dominating the available phase space. Whilst not required for the cogency of their account in quite the same way, the Gibbsian also depends on the thermodynamic limit/large N. For, as mentioned above, Gibbsian ensembles are only equivalent in the thermodynamic limit – a fact held as vital by physicists. For instance, Huang says “From a physical point of view, a microcanonical ensemble must be equivalent to a canonical ensemble, otherwise we would seriously doubt the utility of either” (Huang 1987, p. 148): cited in (Callender 2011, p. 977). Thus, whilst the Gibbsian canonical ensemble is applicable to a one-molecule gas—unlike the Boltzmannian picture—the thermodynamic limit is nonetheless seen as crucial (Thompson 1972).

27 The free energy is $F=-kTlnZ$ where *Z* is the canonical partition function $Z= \Sigma _n exp(-\frac{E_n}{kT})$. In order for there to be a non analyticity in this function, $n\rightarrow \infty$.

28 Of course, one might independently worry about this for the Gibbs and the Boltzmann entropy.
